# The Effect of Font Size on Children’s Memory and Metamemory

**DOI:** 10.3389/fpsyg.2018.01577

**Published:** 2018-08-28

**Authors:** Vered Halamish, Hila Nachman, Tami Katzir

**Affiliations:** ^1^School of Education, Bar-Ilan University, Ramat Gan, Israel; ^2^Edmond J. Safra Brain Research Center for the Study of Learning Disabilities and Department of Learning Disabilities and Special Education, University of Haifa, Haifa, Israel

**Keywords:** children, font size, judgment of learning, memory, metamemory

## Abstract

Recently, there has been a growing interest in the effect of perceptual features of learning materials on adults’ memory and metamemory. Previous studies consistently have found that adults use font size as a cue when monitoring their learning, judging that they will remember large font size words better than small font size words. Most studies have not demonstrated a significant effect of font size on adults’ memory, but a recent meta-analysis of these studies revealed a subtle memory advantage for large font words. The current study extended this investigation to elementary school children. First and fifth–sixth graders studied words for a free recall test presented in either large or small font and made judgments of learning (JOLs) for each word. As did adults, children predicted they would remember large font size words better than small font size words and, in fact, actually remembered the large font size words better. No differences were observed between the two age groups in the effect of font size on memory or metamemory. These results suggest that the use of font size as a cue when monitoring one’s own learning is robust across the life span and, further, that this cue has at least some validity.

## Introduction

To self-regulate learning effectively, learners need to accurately judge the extent to which the studied information has thus far been learned. However, learners do not have direct access to memory traces, but rather infer the state of learning from various cues such as the characteristics of the learning materials, the conditions of learning, or the subjective experience during learning (Koriat, 1997). Judgments of learning (JOLs; Rhodes, 2016) are predictive of learning only when they rely on cues that are predictive of learning.

One set of cues for JOLs that has received recent attention involve the perceptual features of textual materials. Textual materials can be presented in formats that are more perceptually clear in terms of font size, type, and contrast (e.g., 12-point black Times New Roman font on a white background) or in formats that are more perceptually degraded (e.g., nine-point gray Monotype Corsiva font on a white background). Recent studies have consistently demonstrated that adult learners use such perceptual features as a basis for self-judgments about memory, decision-making and reasoning (e.g., Rhodes and Castel, 2008; Alter and Oppenheimer, 2009; Yue et al., 2013; Pieger et al., 2016). Specifically, perceptually clear materials are judged to be better learned than perceptually degraded materials. There is inconsistency, however, regarding whether such perceptual features are indeed predictive of learning.

Despite the recent growing interest in the effect of perceptual features on adults’ cognition and metacognition, similar effects with children have largely been neglected. Whereas preliminarystudies (French et al., 2013; Katzir et al., 2013; Miele et al., 2013) examined the effect of perceptual features on children’s comprehension and recall of texts, in the current research we focused on memory for isolated words. More specifically, we examined whether the font size of to-be-remembered words would affect children’s memory and metamemory judgments.

### Perceptual Features and Adults’ Metamemory

The perceptual features in which learning materials are perceived or processed have been found to affect adult learners’ predictions about how well they learned these materials. Adults judge that they would remember perceptually clear materials better than perceptually degraded materials. For example, several studies (e.g., Rhodes and Castel, 2008; Kornell et al., 2011; Yue et al., 2013; Mueller et al., 2014) have demonstrated, quite consistently, that to-be-recalled words presented in large or clear fonts are predicted to be better remembered than words that are presented in small or unclear fonts (for a recent meta-analysis, see Luna et al., 2017; but see Miele and Molden, 2010; Miele et al., 2011). It is still debatable whether the higher JOLs for items presented in larger fonts are based on the subjective, mnemonic experience of relatively greater fluency (Rhodes and Castel, 2008; Undorf et al., 2017) or on a more general metacognitive belief that larger fonts enhance memory (Mueller et al., 2014; Hu et al., 2015). Nevertheless, it is clear that adults use font size as a cue when predicting subsequent memory. But is it a valid cue?

### Perceptual Features and Adults’ Memory

Do the perceptual features of learning materials affect adults’ actual learning outcomes? The principle of desirable difficulties (Bjork, 1994; Bjork and Bjork, 2011), which is based on decades of research in cognitive psychology, suggests that conditions that make learning more difficult and challenging for the learner can enhance long-term memory and learning outcomes. According to this principle, designing learning materials that are perceptually more difficult to read might enhance learning. In contrast, cognitive load theory (Sweller et al., 1998, 2011) suggests that learning materials should be presented such that unnecessary demands (i.e., extraneous processing) on our limited processing resources are avoided, in order to leave enough resources for goal-relevant processes (i.e., intrinsic and germane processing).

One line of research focused on the consequences of perceptual features of textual learning materials. Some studies suggested, quite counterintuitively, that perceptually degraded (vs. clear) textual materials enhance adults’ learning (Diemand-Yauman et al., 2011; Experiment 1 in Eitel et al., 2014; Lehmann et al., 2016). For example, Diemand-Yauman et al. (2011) observed greater recall of and learning from textual materials that were presented in a smaller, non-standard, gray font (versus larger, standard, black font).

Yet, other studies failed to replicate these findings. Some studies suggested that perceptual degradation impair, rather than enhance, adults’ cognitive performance (e.g., Lonsdale et al., 2006; Miele and Molden, 2010; Experiment 2a in Yue et al., 2013). For example, Lonsdale et al. (2006) observed that presenting texts in less legible display formats impeded adults’ ability to locate information in these texts. Similarly, Miele and Molden (2010, Experiment 3) observed that non-standard font style reduced adults’ text comprehension. Other studies revealed no effect of perceptual degradation of textual materials on learning outcomes (e.g., Eitel and Kühl, 2016; Experiments 2–4 in Eitel et al., 2014; Pieger et al., 2016; Rummer et al., 2016; Experiments 1a, 1b, 2b, 3 in Yue et al., 2013). Recently, a meta-analysis over these studies found an overall null effect of perceptual degradation of textual materials on learning outcomes (Xie et al., 2018).

In an attempt to provide an explanation that will account for the inconsistent results, Weissgerber and Reinhard (2017) recently argued that degraded textual materials improve learning outcomes on delayed tests but not on immediate tests, and provided evidence supporting this suggestion. Yet much more research is needed to understand the generalizability, boundary conditions and moderators of the effect of perceptually degraded (vs. clear) materials on text-based learning (Eitel et al., 2014; Oppenheimer and Alter, 2014; Dunlosky and Mueller, 2016).

Another line of research, which is more relevant to the current study, focused on perceptual features of to-be-remembered words or word pairs. Following Rhodes and Castel (2008), a common manipulation involved varying the font size of the words, usually 48-point versus 18-point font. Up until recently, studies in this line of research have almost consistently demonstrated that font size of words has no effect on adults’ memory for these words (Rhodes and Castel, 2008; Kornell et al., 2011; Miele et al., 2011; McDonough and Gallo, 2012; Susser et al., 2013; Mueller et al., 2014; Hu et al., 2015). In light of these findings, the use of font size as a cue to predict memory has been interpreted as reflecting a metamemory illusion (Rhodes and Castel, 2008).

However, a recent meta-analysis over these studies (Luna et al., 2017) suggested that there is nevertheless a subtle memory advantage for the larger font words, although this benefit is smaller than predicted by the participants in their judgments of learning. The small mnemonic benefit of the larger font words was also reported in few other recent studies (Price et al., 2016; Halamish, 2018; Undorf and Zimdahl, 2018). This evidence leads to a reinterpretation of the use of font size as a cue when predicting subsequent memory. Rather than reflecting a memory illusion, it appears to reflect a mismatched effect of font size on memory and metamemory. The current research extended previous studies by examining the effect of font size on memory and metamemory for words with elementary school children.

### Perceptual Features and Children’s Reading, Memory, and Comprehension

Despite the recent growing interest in the effect of perceptual features on learning and metacognition in adults, little research on this matter has been conducted with children. This is unfortunate given the potential practical implications of such research for education.

Reading skills develop during elementary school years (Perfetti, 1995; Katzir et al., 2006). During the initial stages of reading in the first and second grades, reading is slow and laborious, and readers are “glued to the print” (Chall, 1991), receiving many cues about how to decode words from the letters themselves. With experience, children develop the ability to use context and meaning to decipher words and reading becomes more fluent and effortless. This developmental shift has been referred to as a shift from learning to read to reading in order to learn (Chall, 1991).

A number of studies (e.g., Tinker, 1963; Watts and Nisbett, 1974; Walker, 1992; Wilkins, 1995) examined whether perceptual features of texts affect reading rate and accuracy. These studies suggest that adequate perceptual analysis is required for proper reading and that it can be enhanced by clear text design. Studies focusing on individual differences found that, for young and struggling readers, larger fonts can enhance reading rate and accuracy (Hughes and Wilkins, 2002; O’Brien et al., 2005; see similar findings about adults, Reber and Schwarz, 1999; Werth and Strack, 2003; Lonsdale et al., 2006).

However, little is known about whether and how perceptual features affect children’s cognitive performance beyond reading (e.g., memory and comprehension). If clear perceptual properties enhance reading accuracy and efficiency, they should leave young readers more cognitive resources and time for elaborated processing than degraded perceptual features, which in turn should enhance subsequent recall and comprehension. Only a few studies have thus far examined the effect of perceptual properties on children’s learning, and their results have been inconsistent. Katzir et al. (2013) observed that smaller than standard font size impaired reading comprehension of second grade children, but improved reading comprehension of fifth grade children. In another study, French et al. (2013) observed that 9th to 11th graders better recalled texts presented in a difficult to read font than in a standard font. Furthermore, they observed that dyslexic children also benefited from reading in a non-standard font, and to a greater degree than non-dyslexic children. These two studies suggest, then, that perceptually degraded materials might have a positive effect on children’s cognitive performance in terms of recall or comprehension, and that such effects might depend on the reader’s level of development or ability (cf. Thompson et al., 2013). In contrast, a study by Miele et al. (2013) suggests that perceptually degraded texts might have a negative effect on children’s comprehension. They observed better reading comprehension of third and fifth graders when texts were presented in a clear font (in terms of font type and brightness) than in an unclear font.

### Perceptual Features and Children’s Metacognition

It is also unclear whether and how perceptual features affect children’s metacognition. In general, research that examined children’s metacognition suggest that schoolchildren can effectively monitor their learning under certain circumstances and that monitoring accuracy develops with age (Koriat and Shitzer-Reichert, 2002; Schneider and Löffler, 2016). Koriat et al. (2009) observed that, as adults, third to sixth graders relied on the mnemonic cue of study time in monitoring their learning, but first and second graders did not. Both age groups demonstrated the belief that recall should tend to increase with study time and, indeed, study time was a valid cue for remembering for both age groups. These findings suggest that the use of mnemonic cues and beliefs when monitoring one’s own learning increase with age.

Do children use font size as a cue when monitoring their learning? The Miele et al. (2013) study, reported above, examined how perceptual features affect children’s meta-comprehension. After reading clear or degraded texts, children reported higher comprehension for the clear text versions than for the unclear versions (but also observed a moderation by naïve theories of intelligence; Dweck, 1999). To the best of our knowledge, there are no other studies that examined the effect of perceptual features on children’s metacognition.

In sum, there is ample evidence that perceptually clear materials enhance children’s reading rate and accuracy. However, the scarce studies that examined the effect of perceptual features on children’s cognitive performance in terms of recall or comprehension provided mixed results. Even less is known about the effect of perceptual features on children’s metacognition. Do children relay on font size as a cue when monitoring their own learning? Clearly, more research is needed before any strong conclusions can be made. The current research takes one step toward this end.

### The Current Research

The purpose of the current research was to examine the effect of font size of written words on memory and metamemory in elementary school children. We examined both younger (first grade) and older (fifth–sixth grade) children. The design and procedure were based on Rhodes and Castel’s (2008) study, with a few modifications to adjust the method for children (i.e., fewer words, longer presentation time, shorter retention interval, age-appropriate JOL scale, and oral response format). Participants studied words written in either a large or a small font and gave a JOL, estimating the chance they would later be able to recall these words, and then took a free-recall memory test.

## Materials and Methods

### Participants

Participants were 59 first graders and 46 fifth and sixth graders from a public school in Israel, with a population of predominantly moderate to high socioeconomic status. All children were native Hebrew speakers, had no vision problems, and no documented history of learning disabilities. All were tested individually toward the end of the academic year, between April and June. Three first graders and two fifth–sixth graders who scored two standard divisions above or below the mean in at least one of the baseline tests were excluded from the analyses, resulting in a final sample of 56 first graders (28 male; age range 6–7.1 years, mean age 6.9 years) and 44 fifth–sixth graders (18 male; age range 10–12.2 years, mean age 11.1 years).

### Materials and Procedure

The study was approved by the Chief Scientist of the Ministry of Education in Israel as well as the Research Ethics Committee of the Department of Education at the University of Haifa. Before testing, written informed consent was obtained from the parents of the participants. Participants were tested individually in a quiet room. In the first session, they completed standardized tests (real words and non-words reading rate and accuracy, Shany et al., 2006; forward and backward digit span, Wechsler, 1998) as baseline measures to ensure they could read normally.

In the second session, participants completed the main task. Materials for this phase consisted of two age-appropriate lists of 24 Hebrew words each, one for each age group, taken from norms (Morag, 2010, Unpublished; see also Dotan and Katzir, 2018; Hadad et al., 2018). All words were two-syllables, 3–5 letter nouns with relatively high frequency (4 or 5 on a 1–5 scale, as rated by 10 elementary school teachers; Morag, 2010, Unpublished). Each list was randomly divided into two sets of 12 items each, which were also matched on familiarity and number of letters.

Participants were seated at a chair placed approximately 80 cm (31 inches; measured from the back of the chair) from a 14-inch computer screen. They were asked to read aloud and study words for a later memory test and were informed that the words would be displayed in various font sizes. The 24 words were then presented one at a time on a computer screen for 8 s each in black David font on a white background. Words from one set were presented in a 48-point font and words from the other set were presented in an 18-point font (actual sizes of these fonts on the 14-inch screen were 9.5 and 3.5 mm high for the standard-size Hebrew letter “⊃” for the large and small font size, respectively). The assignment of font size to sets was counterbalanced across participants. Words from the two sets were presented in alternation. The first and last words (one in a small font size and the other in a large font size, counterbalanced across participants), served as primacy and recency buffers, and were excluded from all analyses.

Words for fifth–sixth graders were presented either with or without diacritic marks—the two Hebrew orthography formats that children at this age are used to reading—between participants. We had no specific prediction regarding the role of diacritic marks on memory or metamemory performance. The results below are therefore reported collapsed across these two versions. (For the sake of completeness, results for the effect of diacritic marks are reported in the **Appendix**.)

**FIGURE 1:**
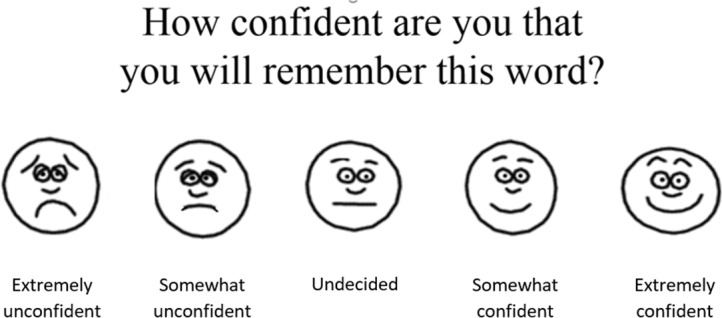
The JOL scale.

Immediately following the presentation of each word, participants provided a JOL. They were prompted to estimate the chance that they would later be able to recall that word, on a child-friendly five-point scale adopted from Kasperski and Katzir (2013). This scale consisted of five facial expressions ranging from happy to sad, and labeled *extremely confident*, *somewhat confident*, *hesitant*, *somewhat unconfident*, and *extremely unconfident* (see **Figure 1**). Participants had 8 s to provide their JOL by pointing at the appropriate facial expression, and the experimenter recorded their response.

Immediately following the presentation of the study list, participants engaged in a filler task for 30 s, solving a maze. Then they were asked to freely recall out loud as many list words as they could while the experimenter recorded their responses.

## Results

**FIGURE 2:**
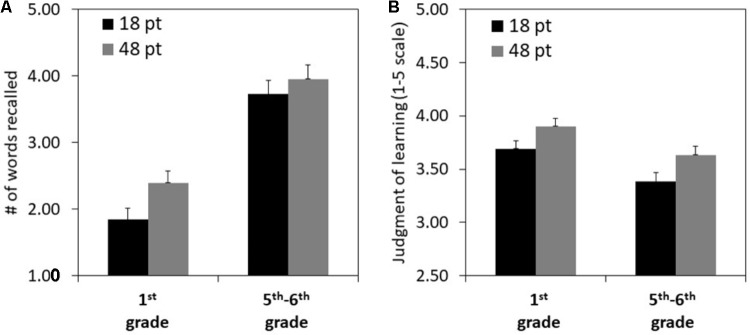
Mean number of words recalled **(A)** and judgments of learning **(B)** by age group and font size. Error bars represent 1 standard error of the mean.

All children included in the final sample were able to read aloud all the words in the main task. On the test, there were relatively few intrusions (i.e., output of a non-studied word; *M* = 0.20, *SD* = 0.57, *95% CI* [0.09, 0.31]). The number of intrusions did not differ by age group, *t*(98) = 0.99, Cohen’s *d* = 0.20. **Figure 2** presents the means and standard errors of the JOLs and number of words correctly recalled by age group and font size (see **Supplementary Table S1** for the raw data). We conducted two-way mixed analyses of variance on each of the dependent measures reported below, with age group as a between-subjects factor and font size as a within-subjects factor.

### The Effect of Font Size on Memory

First, we examined the effect of font size and age group on memory performance in terms of the number of recalled words. Results revealed a significant main effect of age group. Fifth–sixth graders correctly recalled significantly more words per subset (*M* = 3.84, *SD* = 1.07, *95% CI* [3.55, 4.13]) than first graders (*M* = 2.12, *SD* = 0.87, *95% CI* [1.86, 2.37]), *F*(1,98) = 78.61, *MSE* = 1.87, *p* < 0.001, ${\text{η}}_{\text{p}}^{2}$ = 0.45. Results further revealed a small but significant main effect of font size. Participants correctly recalled more large font words (*M* = 3.08, *SD* = 1.56, *95% CI* [2.77, 3.39]) than small font words (*M* = 2.67, *SD* = 1.63, *95% CI* [2.35, 2.99]), *F*(1,98) = 4.26, *MSE* = 1.76, *p* = 0.042, ${\text{η}}_{\text{p}}^{2}$ = 0.04. These results suggest that, for children, larger font size enhances memory. The interaction of age group and font size was not significant, *F*(1,98) = 0.74, *MSE* = 0.01, *p* = 0.390.

### The Effect of Font Size on Metamemory Judgments

Next, we examined the effect of font size and age group on JOLs. Results revealed a significant main effect of age group. First graders gave significantly higher JOLs (*M* = 3.80, *SD* = 0.55, *95% CI* [3.67, 3.93]) than fifth–sixth graders (*M* = 3.51, *SD* = 0.41, *95% CI* [3.36, 3.66]), *F*(1,98) = 8.51, *MSE* = 0.49, *p* = 0.004, ${\text{η}}_{\text{p}}^{2}$ = 0.08. This finding suggests that first graders were more confident in their ability to remember words than the fifth–sixth graders, despite actual lower recall performance. Importantly, results further revealed a significant main effect of font size. Participants gave higher JOLs for large font words (*M* = 3.78, *SD* = 0.58, *95% CI* [3.67, 3.90]) than for small font words (*M* = 3.56, *SD* = 0.59, *95% CI* [3.44, 3.67]), *F*(1,98) = 16.02, *MSE* = 0.16, *p* < 0.001, ${\text{η}}_{\text{p}}^{2}$ = 0.14. The interaction of age group and font size was not significant, *F*(1,98) = 0.14, *MSE* = 0.16, *p* = 0.713. These results suggest that children, just as adults in previous studies, used font size as a cue and estimated they would recall large font words better than small font words.

### The Effect of Font Size on Metamemory Accuracy

Although beyond the central purpose of the current study, we also examined the effect of font size on JOLs accuracy in terms of resolution (see Susser et al., 2013; Yue et al., 2013 for similar analyses with perceptual manipulations). Resolution is the relative correspondence between the predicted (JOLs) and actual memory performance. It reflects the ability of participants to distinguish in their JOLs between words they would recall and words they would not remember. Resolution was examined in terms of the within-participants Goodman–Kruskal gamma correlations between JOLs and whether a word was recalled or not. Eleven first graders and one fifth–sixth grader were excluded from the analysis because they had no variance for either JOLs or memory performance for at least one of the font sizes. We examined the effect of font size and age group on these correlations. Results revealed that the main effect of age group was not significant, *F*(1,86) = 0.68, *MSE* = 0.43, *p* = 0.413, suggesting equivalent resolution for first (*M* = 0.29, *SD* = 0.55, *95% CI* [0.15, 0.42]) and fifth–sixth (*M* = 0.37, *SD* = 0.36, *95% CI* [0.23, 0.51]) graders. Gamma correlations were somewhat higher for the large-font words (*M* = 0.40, *SD* = 0.57, *95% CI* [0.28, 0.52]) than for the small-font words (*M* = 0.25, *SD* = 0.63, *95% CI* [0.12, 0.39]), but this difference was not significant, *F*(1,86) = 3.09, *MSE* = 0.30, *p* = 0.083. The interaction of age group and font size was not significant, *F*(1,86) = 0.36, *MSE* = 0.30, *p* = 0.552.

## Discussion

In recent years, there has been a growing interest in the effect of perceptual features of written materials in general, and font size in particular, on adults’ memory and metamemory. As far as we know, the current experiment was the first to examine the effect of font size on memory and metamemory in children. The results suggest that overall, first graders remembered fewer words yet gave higher JOLs than the fifth–sixth graders. Importantly, the children predicted they would remember large font size words better than small font size words, and they actually remembered the large font size words slightly better. Age group did not interact with the effect of font size on either memory or metamemory.

The effect of font size on children’s JOLs was relatively large. It is consistent with the findings for adults that have been repeatedly replicated since the original study by Rhodes and Castel (2008; see Luna et al., 2017 for a meta-analysis). The finding that children’s memory was indeed better for larger than for smaller fonts was relatively small, which is consistent with the subtle effect of font size on adults’ memory, as revealed in recent meta-analyses (Luna et al., 2017; Halamish, in press; see also Undorf and Zimdahl, 2018^1^), although it was not observed in most individual studies that reported a null effect (e.g., Rhodes and Castel, 2008). Luna et al. interpreted their results as suggesting that the effect of font size on adults’ metamemory does not reflect a complete metamemory illusion but rather a mismatched effect of font size on memory and metamemory. In other words, font size does not reflect a case in which metamemory relies on cues that are not predictive of learning, but rather a case in which metamemory relies on cues that are predictive of learning but does so disproportionally. The current results support this interpretation and extend it to elementary school children.

Interestingly, both the younger and the older children relied on font size as a cue, and to a similar extent, despite developmental trends in the use of other cues (Koriat et al., 2009). Together with the finding that font size is consistently used as a cue by young adults (Luna et al., 2017) as well as older adults (Price et al., 2016), the results point to the robustness of use of this cue when monitoring learning across the life span. Whether children’s use of font size as a cue emerges from reliance on subjective experience of greater fluency when processing larger words or on metacognitive beliefs that larger words are easier to remember is still an open question, as it is for adults (Mueller et al., 2014; Hu et al., 2015; Undorf et al., 2017), and awaits further research.

The finding that larger font size enhanced children’s memory, though subtle, is consistent with the idea that difficulties are not always desirable (Bjork and Bjork, 2011; Yue et al., 2013). To use the terminology suggested by cognitive load theory (Sweller et al., 1998, 2011), for children, memory might be weaker for small font words because relatively small font size creates extraneous load that impairs cognitive processes supporting better memory. However, one should keep in mind that cognitive load theory was usually applied to relatively complex learning materials, whereas the current study demonstrates the idea with more simple stimuli of single words.

It is interesting to compare the current results regarding the effect of font size on children’s memory to the prior findings (Katzir et al., 2013) on the effect of font size on children’s functioning. For younger children (first-second graders), larger font size enhanced both memory (in the current study) and reading comprehension (in the 2013 study). These results can be explained by the demands of the early stages of reading acquisition, when readers are “glued to the print” (Chall, 1991) and rely heavily on the letters themselves to decode words. These results support the need for and the often-used practice of using larger fonts sizes in instructional materials for younger children, not only for the sake of reading *per se*, but also for the sake of learning outcomes. For older children (fifth–sixth graders), larger font size enhanced memory (in the current study) but impaired reading comprehension (in the 2013 study). Therefore, the effect of font size on learning might depend not only on age, but also on the criterion task or the goal of learning (McDaniel and Butler, 2011). However, caution is needed when comparing the results of the two studies because the actual font sizes were different (i.e., they were overall smaller in the study by Katzir et al., 2013 than in the current study).

The current research was the first to examine the effect of font size on children’s memory and metamemory. Of course, few issues await future research. First, the task used in the current study was built after the common method in previous font size experiments (e.g., Rhodes and Castel, 2008), but some parameters were modified to adapt the task to the use with children (e.g., fewer words, longer presentation time). Future research might examine whether the similar effects of font size on memory and metamemory for adults (in previous studies) and children (in the current study) persist when the same parameters are used for adults and children. Second, future research could examine whether font size affects children’s recognition (rather than recall) memory, as been recently demonstrated for adults (Halamish, 2018), especially given the overall low free-recall rates for children in the current study. Third, future research could examine whether the effect of font size on children’s JOLs is reduced when JOLs are delayed, as was recently demonstrated for adults (Luna et al., 2017).

Finally, another avenue for future research could be to examine the effect of more extreme font sizes on children’s memory and metamemory. Using adult participants, Halamish (in press) and Undorf and Zimdahl (2018) recently observed that under certain conditions, not only large but also very small fonts (e.g., five-point) enhance memory for words. These findings are consistent with prior scarce evidence that substantial manipulations of perceptual degradation enhance adults’ memory (Hirshman and Mulligan, 1991; Sungkhasettee et al., 2011). Together, these studies emphasize that the strength of the perceptual manipulation might moderate its effect on memory and that is should be considered in future research with adults and children alike.

## Conclusion

The current research suggests that, as adults, children use font size as a cue when monitoring their own learning and predict better memory for larger font words, and that they indeed remember larger font words slightly better. Pedagogical tools developed for children might take into consideration the potential positive, thought relatively subtle, effect of larger print on learning. More research is needed, however, to examine whether the effect generalizes to other populations (e.g., children with learning disabilities rather than normal readers), other presentation formats (e.g., on paper rather than on screen), and more complex learning materials (texts rather than words). Yet, the current results provide the interesting insight that for children, small, technical changes in display format of textual materials results in meaningful effects on both metacognition and memory.

## Author Contributions

VH, HN, and TK conceived and designed the experiment and contributed to writing the manuscript. HN conducted the experiment. VH and HN analyzed the data.

## Conflict of Interest Statement

The authors declare that the research was conducted in the absence of any commercial or financial relationships that could be construed as a potential conflict of interest.

## Footnotes

1 For the sake of transparency, we note that these studies were published after we conducted and analyzed the data of the current study.

## APPENDIX

### The Effect of Diacritic Marks on Fifth–Sixth Graders’ Memory and Metamemory Judgments

Hebrew orthography includes two formats. One is a more “shallow” format in which words are written with diacritic marks and there is a direct and systematic relation between the consonants in the alphabet and their pronunciation. The second is a more “deep” format in which words are written without diacritic marks and the relation between consonants and their pronunciation is less consistent. Young children learning to read, as were the first graders in the current experiment, learn from materials with diacritic marks. We therefore included such diacritic marks in the word presentation for this age group. As kids grow older, they gradually transfer from third grade to reading materials without diacritic marks. We therefore had two versions of word presentation for the fifth–sixth graders, one with and one without diacritic marks. Fifth–sixth graders were randomly assigned to the diacritics (*N* = 23) and the no-diacritics (*N* = 21) conditions. The effect of diacritic marks on memory or metamemory was outside the focus of the reported study and we had no specific prediction for it. Results for this variable are reported here for the sake of completeness.

We conducted two-way mixed analyses of variance with diacritic marks as a between subjects factor and font size as a within subjects factor on both memory and JOLs. Analysis of the number of recalled words did not reveal a significant effect of diacritic marks, *F*(1,42) = 0.08, *MSE* = 2.34, *p* = 0.782; nor was there a significant interaction between font size and diacritic marks, *F*(1,42) = 2.75, *MSE* = 1.65, *p* = 0.104. Analysis of the JOLs did not reveal a significant effect of diacritic marks, *F*(1,42) = 2.07, *MSE* = 0.33, *p* = 0.158. The analysis did yield a significant interaction between font size and diacritic marks, *F*(1,42) = 4.64, *MSE* = 0.08, *p* = 0.037, ${\text{η}}_{\text{p}}^{2}$ = 0.10. Further analyses revealed that, for the small font size, participants gave lower JOLs with diacritic marks (3.23) than without them (3.53), *t*(42) = 2.13, *p* = 0.040, Cohen’s *d* = 0.66. However, for large font size, diacritic marks did not affect JOLs (3.61 and 3.66 with and without diacritic marks, respectively, *t*(42) = 0.39, *p* = 0.702).
